# MR Imaging Features of Gadofluorine-Labeled Matrix-Associated Stem Cell Implants in Cartilage Defects

**DOI:** 10.1371/journal.pone.0049971

**Published:** 2012-12-12

**Authors:** Hossein Nejadnik, Tobias D. Henning, Thuy Do, Elizabeth J. Sutton, Frederick Baehner, Andrew Horvai, Barbara Sennino, Donald McDonald, Reinhard Meier, Bernd Misselwitz, Thomas M. Link, Heike E. Daldrup-Link

**Affiliations:** 1 Department of Radiology, Stanford University, Stanford, California, United States of America; 2 Department of Diagnostic Radiology, University Hospital of Cologne, Cologne, Germany; 3 Department of Radiology, Mt Auburn Hospital, Boston, Massachusetts, United States of America; 4 Department of Pathology, UCSF Cancer Center, University of California San Francisco, San Francisco, California, United States of America; 5 Department of Anatomy and CVRI, University of California San Francisco, San Francisco, California, United States of America; 6 Research Laboratories of Bayer Schering Pharma AG, Berlin, Germany; 7 Department of Radiology, UCSF Medical Center, University of California San Francisco, San Francisco, California, United States of America; Instituto de Engenharia Biomédica, University of Porto, Portugal

## Abstract

**Objectives:**

The purpose of our study was to assess the chondrogenic potential and the MR signal effects of *GadofluorineM-Cy* labeled matrix associated stem cell implants (MASI) in pig knee specimen.

**Materials and Methods:**

Human mesenchymal stem cells (hMSCs) were labeled with the micelle-based contrast agent *GadofluorineM-Cy*. Ferucarbotran-labeled hMSCs, non-labeled hMSCs and scaffold only served as controls. Chondrogenic differentiation was induced and gene expression and histologic evaluation were performed. The proportions of spindle-shaped vs. round cells of chondrogenic pellets were compared between experimental groups using the Fisher's exact test. Labeled and unlabeled hMSCs and chondrocytes in scaffolds were implanted into cartilage defects of porcine femoral condyles and underwent MR imaging with T1- and T2-weighted SE and GE sequences. Contrast-to-noise ratios (CNR) between implants and adjacent cartilage were determined and analyzed for significant differences between different experimental groups using the Kruskal-Wallis test. Significance was assigned for p<0.017, considering a Bonferroni correction for multiple comparisons.

**Results:**

Collagen type II gene expression levels were not significantly different between different groups (p>0.017). However, hMSC differentiation into chondrocytes was superior for unlabeled and *GadofluorineM-Cy*-labeled cells compared with Ferucarbotran-labeled cells, as evidenced by a significantly higher proportion of spindle cells in chondrogenic pellets (p<0.05). *GadofluorineM-Cy*-labeled hMSCs and chondrocytes showed a positive signal effect on T1-weighted images and a negative signal effect on T2-weighted images while Ferucarbotran-labeled cells provided a negative signal effect on all sequences. CNR data for both *GadofluorineM-Cy*-labeled and Ferucarbotran-labeled hMSCs were significantly different compared to unlabeled control cells on T1-weighted SE and T2*-weighted MR images (p<0.017).

**Conclusion:**

hMSCs can be labeled by simple incubation with *GadofluorineM-Cy.* The labeled cells provide significant MR signal effects and less impaired chondrogenesis compared to Ferucarbotran-labeled hMSCs. Thus, *GadoflurineM-Cy* might represent an alternative MR cell marker to Ferucarbotran, which is not distributed any more in Europe or North America.

## Introduction

New cell-based therapies for osteoarthritis and rheumatoid arthritis are currently being developed with the goal of providing regeneration of bone and cartilage. It has been shown that hyaline cartilage could be remodelled to some extent after autologous implantation of chondrocytes [1], [2] and bone defects could be repaired by implantation of autologous osteoblasts in a calcium phosphate matrix [3]. Chondrocyte implants for cartilage regeneration have entered clinical practice [4]. However, these implants partly tend to form fibrocartilage instead of hyaline cartilage [5] and recovery is slower compared with osteochondral autograft implantation (OAT) [6].

Human mesenchymal stem cells (hMSC) represent another option for joint regeneration. hMSCs are well characterised autologous cells, which are obtained by a bone marrow aspirate and efficiently expanded in vitro [7]. They may differentiate towards osteocytes and chondrocytes and, thereby, may regenerate destructed joint components [8]. Former investigations have shown that hMSC-based joint regeneration requires the use of scaffolds and selective differentiating factors [8], [9], [10]. The differentiation outcomes of hMSCs embedded in biomaterials and in the context of arthritic joints remains to be studied [7], [8], [9], [10], [11].

MR imaging provides a non-invasive means of tracking matrix-associated cell implants in osteochondral defects. Among various available imaging techniques for cell tracking [12], [13], [14], MR imaging has the distinct advantages of providing direct cartilage depiction with high anatomical resolution, high soft tissue contrast and no radiation exposure. In previous studies, stem cells were labeled with superparamagnetic iron oxide nanoparticles (SPIO) for their direct depiction in cartilage defects with MR imaging [15], [16], [17]. SPIO allow for cell labeling by simple incubation. However, SPIO produce a signal void on all pulse sequences which is indistinguishable from postoperative artifacts, SPIO may interfere with the chondrogenesis of hMSC [17], [18] and commercially available Ferucarbotran is only available in Japan, but not any more in Europe or North America. In pursuit of an alternative cell label, we identified several favorable characteristics of the micelle-based gadolinium-chelate *GadofluorineM-Cy: GadofluorineM-Cy* provides cell labeling by simple incubation, positive signal effect on T1-weighted MR scans, no reported disturbances of cell viability or function and allows direct correlations of imaging data with fluorescence microscopy [19], [20], [21].

Thus, the purpose of our study was to assess the chondrogenic potential and the MR signal effects of *GadofluorineM-Cy* labeled matrix-associated stem cell implants (MASI) in pig knee specimen. Non-labeled and SPIO-labeled MASI served as controls.

## Materials and Methods

### Cells culture and labeling

Commercially purchased human mesenchymal stem cells (hMSC, Lonza Walkersville, Inc., Walkersville, MD, USA), were cultured in DMEM-High Glucose medium (Invitrogen, Carlsbad, CA, USA) containing 10% FBS (Hyclone, Logan, UT, USA) and 1% Penicillin-Streptomycin. The purity of the cells was tested by flow cytometry and their differentiation ability into chondrogenic, osteogenic and adipogenic lineages was documented by the provider. Cells tested positive for CD105, CD166, CD29, and CD44 and negative for CD14, CD34 and CD45. All experiments were performed in between passages 8 and 12 of hMSCs to avoid senescence and ensure full chondrogenic potential.

Cells were labeled with *GadofluorineM-Cy* (Bayer Schering AG, Berlin, Germany). *GadofluorineM-Cy* is an amphiphilic gadolinium (Gd) chelate, composed of a Gd-DO3A derivative with a lysine backbone, a hydrophilic sugar moiety (mannose) and a perfluorinated lipophilic side chain [22], [23], [24]. It has an r1-relaxivity of 17.4 mM^−1^ s^−1^ in blood at 1.5 T and 37°C. For this study, a fluorescent dye, 1,1′-Bis(sulfobutyl) indocarbocyanine-5-carboxylic acid, was covalently attached to the lysine backbone, thereby replacing the sugar moiety with a cyanine dye. The resultant *GadofluorineM-Cy* exhibits fluorescence with an excitation peak of 521.9 nm and an emission peak of 569.32 nm. Labeling of hMSCs with *GadofluorineM-Cy* was achieved by simple incubation at a concentration of 11.9 µmol Gd/ml medium for 24 hours.

Control experiments were performed with hMSC, labeled with the SPIO ferucarbotran. Ferucarbotran is composed of an iron oxide core and an anionic carboxydextran coat. It has a mean diameter of 60 nm, an r1-relaxivity of 25 mM^−1^ s^−1^ and an r2-relaxivity of 151 mM^−1^ s^−1^ at 0.47T and 37°C [25]. Labeling of hMSC with Ferucarbotran was achieved by simple incubation with a concentration of 100 µg Fe/ml medium for 18 hours as described previously [26].

After completion of the labeling procedures, the cells were washed three times with PBS. The concentration of Gd and Fe within the labeled hMSCs was determined by inductively coupled plasma atomic emission spectrometry (ICP-AES) (IRIS Advantage, Thermo Jarrell-Ash, MA). Cellular viability was assessed by the MTS-assay according to the manufacturer's directions (Cell Titer 96 AQ, Promega, Madison, WI).

### Chondrogenic differentiation

2.5*10^5^ hMSCs were resuspended in 0.5 ml of complete chondrogenic medium (Lonza) containing 10 ng/ml rTGF-ß3 in 15 ml polypropylene centrifuge tubes (VWR, West Chester, PA, USA). The medium was changed every 3 days and chondrogenic pellets were harvested at day 0, 7, and 14 for gene expression quantification and day 14 for histological evaluation. In a pulse chase experiment to assess the long term labeling stability, the medium was collected every 3 days and examined by spectrometry (ACP-AES) for the amount of iron or gadolinium that was released by the cell pellets.

### Gene expression evaluation

The effect of *GadofluorineM-Cy* and Ferucarbotran-labeling on the chondrogenic differentiation potential of the hMSCs was evaluated by quantification of collagen type II gene expression. hMSC pellets were harvested at day 0, 7, and 14 of chondrogenic differentiation and then subjected to qPCR expression analysis for collagen type II and the control marker GAPDH. Total cellular RNA was extracted from each sample with the QIAGEN RNeasy® mini kit. cDNA was prepared from total RNA and quantitative real-time PCRs (qPCRs) were carried out and analyzed on an Applied Biosystems StepOne™ Real-Time PCR System. The formation of double-stranded DNA product was monitored by TaqMan® gene expression primers. Expression data were collected as Ct values and the gene expression levels were normalized to the reference control gene, GAPDH.

### Histopathology of labeled cells and chondrogenic pellets

Confocal microscopy was used to localize the contrast agent in labeled cells. *GadofluorineM*-*Cy* labeled cells were stained with DAPI alone because *GadofluorineM-Cy* posesses intrinsic fluorescence. Fe-labeled cells were stained with anti-dextran FITC (Stem Cell Technologies, Tukwila, WA, USA) for localization of Ferucarbotran and counterstained with DAPI (Vectashield with DAPI, Vector Laboratories, Burlingame, CA, USA).

Triplicate samples of chondrogenic pellets were fixed in 10% Neutral Buffered Formalin, encapsulated in HistoGel (both Richard-Allan Scientific, Kalamazoo, MI, USA) and stained with Safranin O and Alcian blue. One observer, who was blinded to the experimental groups, counted spindle shaped cells on H&E stains in a representative 100×100 µm field of view at 400× magnification and assigned a semi-quantitative score (0 = no spindle shaped cells, 1 = 1–33%, 2 = 34–66%, 3 = 67–100% spindle shaped cells) [18].

Glucosaminoglycan production was quantified by optical density measurement of the alcian blue stains of the different labeling groups by using Color Deconvolution plugin [27] of ImageJ software (version 1.45s, a free image processing and analysis program developed by National Institutes of Health). Color Deconvolution plugin separated the Alcian blue stain from the counterstain (nuclear fast red). Mean optical density of Alcian blue stains were quantified by converting the mean pixel values of the pellets to optical density by Uncalibrated OD, using the function Unc. OD = log10(255/mean pixel value).

### Scaffold preparation

Pads of Surgifoam (Johnson & Johnson, New Brunswick, NJ, USA), an absorbable gelatin sponge, were immersed in fluid Agarose Type IX Ultra Low (Sigma Aldrich, 1.5% in PBS) at 37°C. Scaffolds were examined by light microscopy for absence of micro bubbles. Then, cells were injected into the scaffold (5*10^5^ per implant) and gelling was induced at 15°C. Scaffolds were cut and implanted in cartilage defects.

### Induction of Cartilage Defects and hMSC implantation

Studies were carried out *ex vivo* in 21 pig knee joint specimens, supplied by a local meat market. A medial skin incision was made, the patella was dislocated laterally and two cubical full thickness cartilage defects (3×3 mm^2^) were created per joint in either femoral condyle. Scaffolds were implanted in 42 created cartilage defects. Experimental groups of six implants each comprised (1) Scaffold only, (2) scaffold with non-labeled undifferentiated cells, (3) scaffold with *GadofluorineM-Cy*-labeled undifferentiated cells, (4) scaffold with Ferucarbotran labeled undifferentiated cells, (5) scaffold with unlabeled chondrocytes (derived from hMSC via the protocol above), (6) scaffold with *GadofluorineM-Cy* labeled chondrocytes and (7) scaffold with Ferucarbotran labeled chondrocytes. Knee joints were closed with surgical sutures after the scaffold implantation.

### Magnetic Resonance Imaging

Immediately after scaffold implantation, all knee joints underwent MR imaging, using a clinical 3 T MR system (Signa EXCITE HD, GE Medical Systems, Milwaukee, WI, USA) and a quadrature wrist coil (Clinical MR Solutions, Brookfield, WI, USA). Sagittal MR images were obtained using a T1 spin echo (SE) sequence (TR 500 ms, TE 15 ms, BW 15.63 Hz, FOV 12 cm, matrix 512×192, 2 acquisitions, acquisition time 3∶16 minutes), a T2 fat-saturated fast spin echo (FSE) sequence (4300/25/31.25/15/512×256/2/4:14, echo train length 9), a T1 3D spoiled gradient recalled echo (3D-SPGR) sequence (17/8.5/16/512×512/0.75/10:44, alpha 12) and a T2* gradient echo (GE) sequence (500/14/15.63/12/512×192/2/3:16, alpha 30). All sequences were acquired with a slice thickness of 1 mm.

All MR images were analyzed using Osirix image processing software (Osirix, UCLA, Los Angeles, CA). Signal intensities (SI) of the different implants, adjacent cartilage and background noise in phase encoding direction were measured with operator-defined regions of interest. The contrast between the cell implants and the adjacent cartilage was quantified as the contrast-to-noise-ratio: CNR = |SI(implant)-SI(cartilage)|/background noise [28].

### Histopathology of implants

After imaging, the implants were removed, fixed and paraffin embedded. 5 µm sections were stained with haematoxylin and eosin (H&E, Sigma Aldrich) and histologically evaluated with a light microscope (Olympus BX-42; Center Valley, PA) for presence and distribution of cells.

### Statistical analyses

A Kruskal-Wallis test, a non-parametric test, was performed to evaluate differences in CNR data and and ANOVA was used for qPCR data of different experimental groups. The Fisher's exact test was used to evaluate differences among semi-quantitative histological scores of three experimental groups (unlabeled, *GadofluorineM-Cy* and Ferucarbotran-labeled hMSCs). A Bonferroni correction was applied for comparisons of multiple experimental groups. A p value of less than 0.017 was considered to indicate significant differences between *GadofluorineM-Cy*-labeled cells, Ferucarbotran-labeled cells and unlabeled cells.

## Results

### Cell labeling

**Figure 1:**
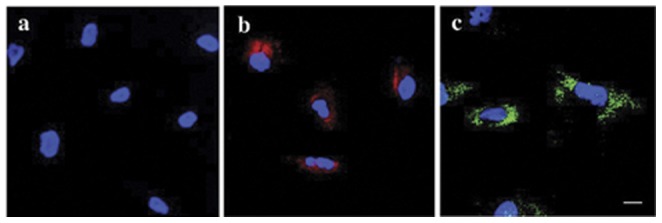
Confocal microscopy. Unlabeled control (A), *GadofluorineM-Cy*-labeled hMSCs (B) and anti-dextran-FITC stain of Ferucarbotran-labeled hMSCs (C). All cells have been counterstained with DAPI (blue). Note the cytoplasmatic localization of both contrast agents (B, C) whereas no contrast agent could be seen in the nucleus. Scale bar = 10 µm.

Inductively coupled plasma atomic emission spectrometry showed significant uptake of *GadofluorineM-Cy* (6.48 pg Gd/cell) and Ferucarbotran (6.26 pg Fe/cell) in hMSC when compared to unlabeled controls (below detection limits for Gd, <0.5 pg Fe/cell for Ferucarbotran). Confocal microscopy confirmed cytoplasmic localization of either contrast agent (Fig. 1). The MTS-assay demonstrated no significant decrease in viability of labeled cells. The median formazan absorbance values were 0.51 for the unlabeled control, 0.48 for *GadofluorineM-Cy* and 0.51 for Ferucarbotran.

### Differentiation of labeled and unlabeled hMSCs

**Figure 2:**
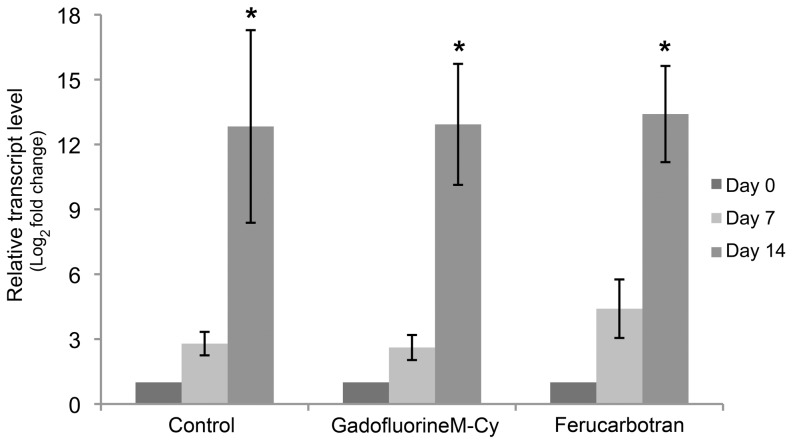
qPCR analysis of collagen type II gene expression. Relative collagen type II mRNA transcript level of unlabeled controls (left columns), *GadofluorineM-Cy*-labeled hMSCs (middle columns) and Ferucarbotran-labeled hMSCs (right columns), displayed as mean data and standard error of triplicate experiments in each group (* indicates significant increase of gene expression of all labeled and unlabeled hMSCs over the time, p value<0.017).

The qPCR analysis of collagen type II gene expression showed an increase of collagen type II gene expression of all labeled and unlabeled hMSCs over the time (p values for control, GadofluorineM-Cy and Ferucarbotran labeled group was 0.011, 0.003, 0.000 respectively). There were no significant differences between the unlabeled, *GadofluorineM-Cy* labeled and Ferucarbotran labeled hMSCs after 14 days of differentiation (p = 0.97) (Fig. 2).

**Figure 3:**
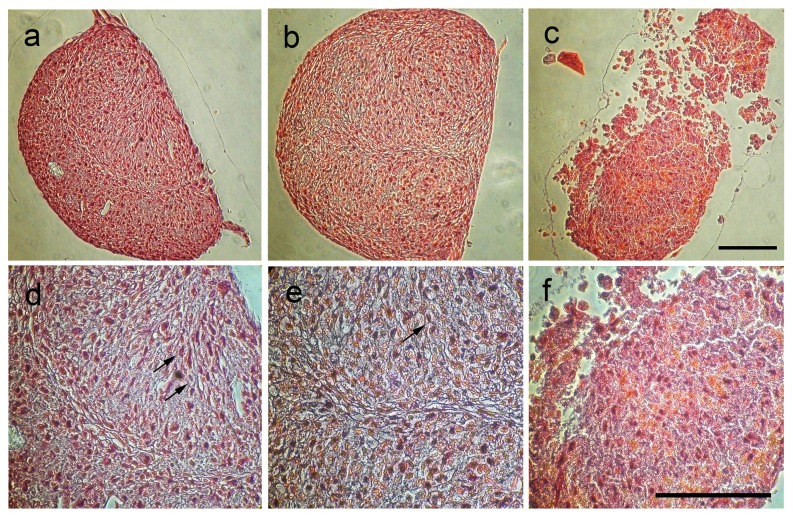
Safranin-O-stains of chondrogenic pellets. Unlabeled control (A and D) and *GadofluorineM-Cy* labeled hMSCs (B and E) show a well-defined histological structure with the formation of spindled cells (black arrows) and intercellular matrix. Ferucarbotran-labeled hMSCs (C and F) show a less regular histological structure and a lower cartilage matrix synthesis (Scale bar = 100 µm).

Safranin-O stains of unlabeled control cells and *GadofluorineM-Cy* labeled cells demonstrated an organized histological architecture consisting of an extracellular cartilage matrix and associated undifferentiated and differentiated mesenchymal cells (Fig. 3). By comparison, Ferucarbotran labeled samples showed islets of extracellular contrast agent, a less organized histological structure and scant amounts of extracellular matrix (Fig. 3). The semiquantitative analysis of TGF-ß3 induced differentiation from round-shaped stem cells to spindle-shaped, differentiated cells revealed a significantly higher proportion of spindle cells in unlabeled and *GadofluorineM-Cy* labeled chondrogenic cell pellets when compared to Ferucarbotran labeled cell pellets (p value = 0.002) (Table 1).

**Table 1:** Semiquantitative scoring of mesenchymal stem cell differentiation from round-shaped into spindle shaped cells.

Labeling protocol	Group 1	Group 2	Group 3
Control	2 (59%)	2 (54%)	2 (63%)
*GadofluorineM-Cy*	2 (53%)	2 (51%)	2 (35%)
Ferucarbotran	1 (29%)	1 (20%)	1 (22%)

Scores
0 = no spindle like cells
1 = 1–33% spindle like cells
2 = 33–66% spindle like cells
3 = 66–100% spindle like cells

**Figure 4:**
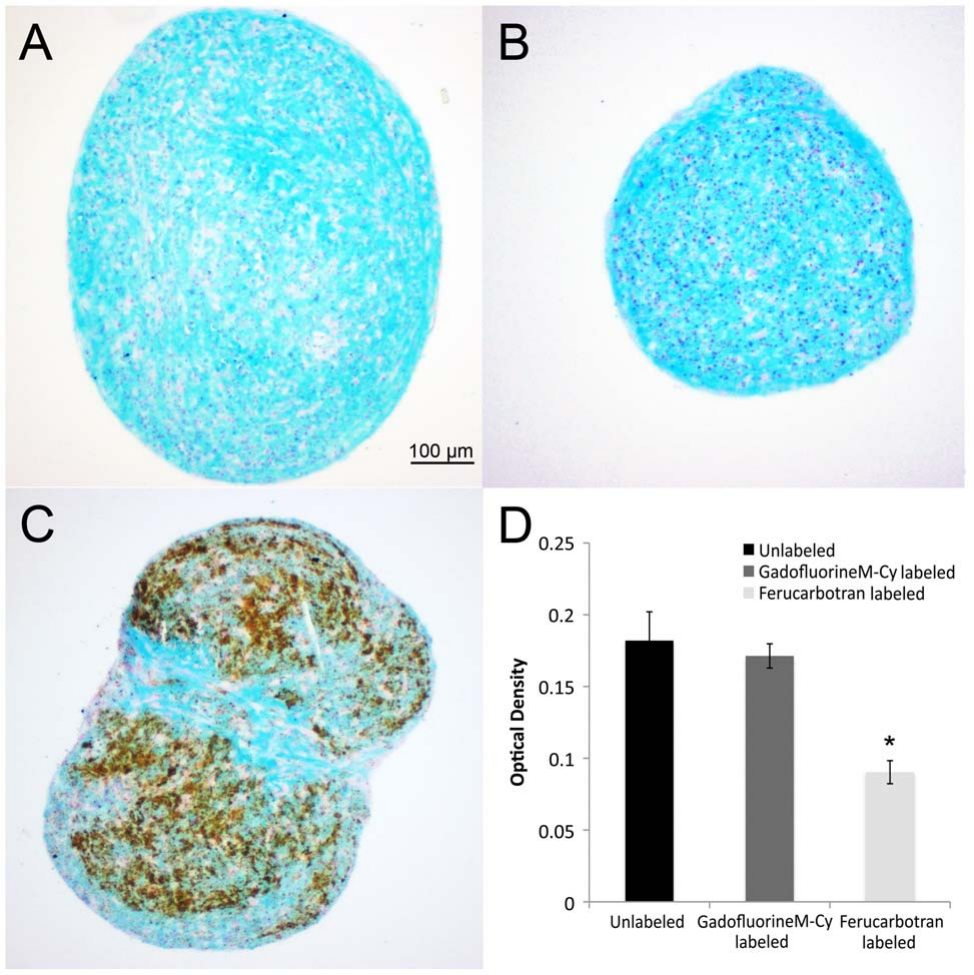
Alcian blue staining of chondrogenic pellets. Unlabeled control (A) and *GadofluorineM-Cy*-labeled pellets (B) show stronger staining for intracellular glycosaminoglycan (GAG) compared to Ferucarbotran-labeled pellets (C). Ferucarbotran-labeled pellets show numerous iron deposits, which can be delineated by their brown color (Scale bar = 100 µm).

In addition, Alcian blue staining, which indicates glycosaminoglycan (GAG) production, was moderate-to-high for unlabeled and *GadofluorineM-Cy* labeled hMSCs, but relatively weak for Ferucarbotran labeled hMSCs (Fig. 4). Optical density measurements of Alcian blue stainings showed a significant difference between Ferucarbotran labeled cells compared to the control and GadofluorineM-Cy groups (p = 0.011).

The pulse chase experiment showed minimal release of either contrast agent over the time period of chondrogenic differentiation. The median amount of released contrast agent compared with the total amount of contrast agent per pellet was 0.009% for *GadofluorineM-Cy* and 0.117% for Ferucarbotran.

### MR imaging of implants in cartilage defects

**Figure 5:**
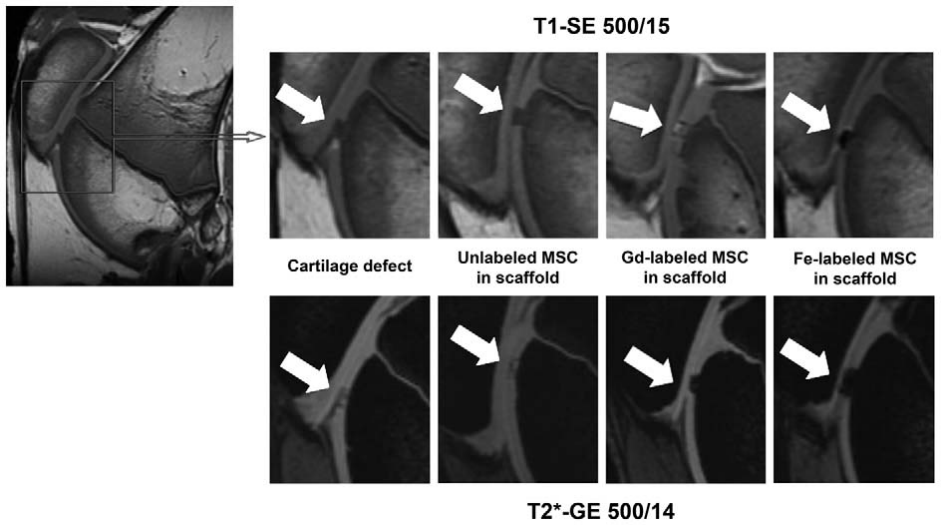
Representative MR images of matrix associated stem cell implants (MASI). Sagittal T1-SE sequences (upper row) and T2* GE sequences (lower row) of cartilage defect, unlabeled MSC in scaffold, Gd-labeled MSC in scaffold, Fe-labeled MSC in scaffold in the femoral condyles (arrows).

**Figure 6:**
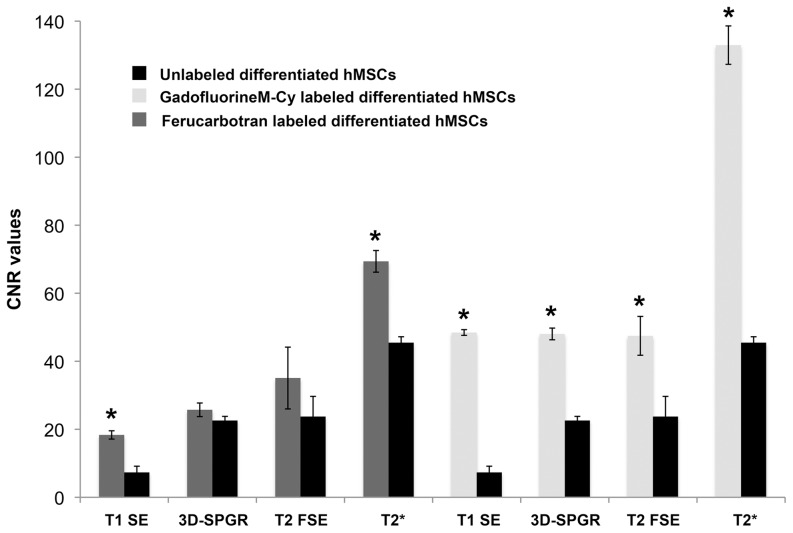
Contrast to noise ratios (CNR) of MASI. CNR values, calculated as the difference in MR signal intensity between labeled MASI and adjacent cartilage for different MR pulse sequences. Data are displayed as mean data of six experiments in each group (*GadofluorineM-Cy*-labeled MASI, Ferucarbotran-labeled MASI and unlabeled controls) with standard deviations (* indicates significant differences between labeled MASI and unlabeled controls, p value<0.017).

Scaffolds without cells and scaffold with unlabeled cells showed no significant difference in MR signal and no significant difference in CNR data on all sequences (Fig. 5, 6). Thus, without labeling, it was impossible to determine if scaffold contained transplanted cells or not. In addition, unlabeled undifferentiated and chondrocyte-differentiated hMSCs showed no significant differences in CNR values on any sequence in either experimental group (unlabeled cells, *GadofluorineM-Cy*-labeled cells, Ferucarbotran-labeled cells).

*GadofluorineM-Cy*-labeled cells (hMSCs or hMSC-derived chondrocytes) showed a moderate T1- and T2-effect (Fig. 5). On T1-weighted MR scans, *GadofluorineM-Cy*-labeled cells appeared isointense or slightly hyperintense compared to adjacent cartilage while scaffold only appeared hypointense compared to adjacent cartilage (Fig. 5). On T2- and T2*-weighted MR images, *GadofluorineM-Cy*-labeled cells appeared hypointense compared to adjacent cartilage. CNR data of *GadofluorineM-Cy*-labeled transplants were significantly different compared to unlabeled control cells on T1-SE (mean CNR = 18.33; p = 0.0039) and T2* GE sequences (mean CNR = 69.37; p = 0.0039). Thus, these sequences could be used to confirm successful implantation of labeled cells within a cartilage defect (Fig. 5, Fig. 6)

Implants of Ferucarbotran-labeled cells appeared hypointense compared to adjacent cartilage on all sequences (Fig. 5). On T2* GE sequences, the size of the Ferucarbotran-induced susceptibility artifact exceeded the size of the cartilage defect, causing a “blooming” effect. CNR data of Ferucarbotran-labeled transplants were significantly different compared to unlabeled control cells on T1-SE (mean CNR = 48.44; p = 0.0039) and T2* GE sequences (mean CNR = 132.96; p = 0.0039). In addition, CNR data of Ferucarbotran-labeled cells were significantly higher compared to *GadofluorineM-Cy*-labeled cells on T1-SE sequences (CNR = 48.44; p = 0.0039) and T2* GE sequences (mean CNR = 132.96; p = 0.0039). (Fig. 6).

**Figure 7:**
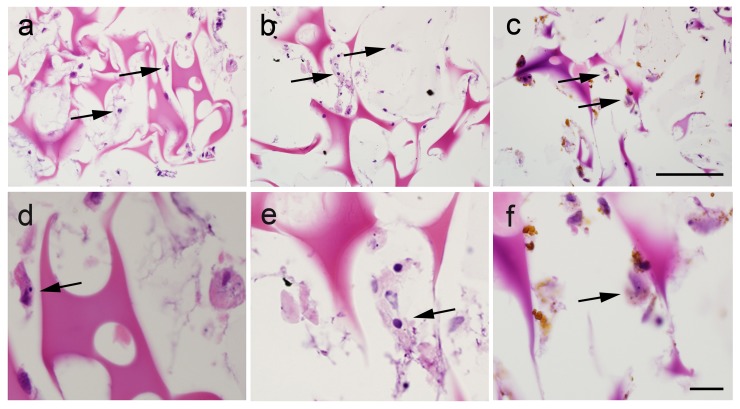
Histology of hMSC implants (H&E staining). All scaffolds show a homogeneous pink staining of the agarose matrix within the gelatin sponge. Injected hMSCs (arrows) can be seen after injection of unlabeled cells (A and D), *GadofluorineM*-*Cy*-labeled cells (B and E) and Ferucarbotran-labeled cells (C and F). While iron oxides can be delineated, *GadofluorineM-Cy* remains invisible at higher magnification and light microscopy (Scale bar = 100 µm).

H&E-stains of scaffolds demonstrated a homogeneous scaffold matrix and structure in all cases (Fig. 7). Presence of hMSCs could be confirmed in all cell-containing scaffolds. Intracellular iron oxides could be delineated in Ferucarbotran-labeled cells (Fig. 7).

## Discussion

To the best of our knowledge, this study represents the first approach of labeling of MASI with the micelle-based Gd-chelate *GadofluorineM-Cy*. *GadofluorineM-Cy* provided significant T2*-signal effect of hMSCs via a simple incubation labeling protocol, without impairment in chondrogenesis of the labeled cells. Thus, *GadoflurineM-Cy* might represent an alternative MR cell marker to ferucarbotran. Ferucarbotran was FDA-aproved in Europe, but is not distributed any more in Europe or North America. The agent is still available in Japan.

Non-invasive depiction of MASI is desirable for monitoring successful outcomes of cartilage regeneration therapies and for diagnosing potential reasons for graft failure, such as uneven distribution of implanted cells within a large defect, cell dislocation into the joint space or cell migration from the implant into adjacent bone marrow [29].

First attempts to visualize matrix-associated hMSC implants in subcutaneous tissues with MR imaging have been obtained with the iron oxide-based contrast agent ferumoxides (Feridex) [29]. A variety of SPIO have been subsequently investigated for the purpose of tracking stem cells in arthritic joints [16], [26]. Our data show that *GadofluorineM-Cy* is advantageous over the SPIO Ferucarbotran for labeling of hMSCs. Even though gene expression results for collagen type II did not show a significant difference, *GadofluorineM-Cy*-labeled hMSCs showed a higher rate of chondrogenic matrix production than Ferucarbotran-labeled cells on Alacian blue stains. This is in accordance with previous reports of an impaired chondrogenesis of iron oxide labeled stem cells [17], [18]. Also, the positive T1 contrast of *GadofluorineM-Cy*-labeled cells is different compared to postoperative susceptibility artefacts caused by air, postsurgical iron depositions or hemorrhage [30]. *GadofluorineM-Cy* has the additional advantage that it can be detected by fluorescence microscopy, thereby facilitating additional arthroscopic optical imaging investigations or histopathological correlations of imaging findings.

Our selection of investigated pulse sequences for depiction of *GadofluorineM-Cy* labeled MASI was based on practical considerations: T1-SE and T2* GE sequences have been previously applied for cell tracking studies [20], [31] and 3D-SPGR and T2-FSE sequences are standard clinical sequences for cartilage imaging [32], [33], [34]. Our data showed, that the T2* GE sequence provides the highest contrast between *GadofluorineM-Cy*-labeled cell implants and adjacent cartilage defects and is therefore best suited for detection of transplanted stem cells.

Other investigators described gadofluorine labeling of hMSCs [19] in vitro and our own group utilized *GadofluorineM-Cy* previously for labeling of monocytes [20]. The *GadofluorineM-Cy* label remained stable for at least 7 days in previous studies [20]. This is in accordance with our pulse chase experiment, which showed no significant release of intracellular contrast agent during chondrogenic differentiation and no significant difference in MR signal of hMSCs and hMSC-derived chondrocytes.

We recognize several limitations of our study: We investigated the chondrogenesis of *GadofluorineM-Cy*-labeled hMSCs *in vitro*. Further studies have to confirm for other stem cell types, such as embryonic stem cells and induced pluripotent stem cells, and for *in vivo* applications, that *GadofluorineM-Cy* does not significantly impair chondrogenesis. Future studies will have to specifically address the biocompatibility of *GadofluorineM-Cy*-labeling techniques *in vivo* and will have to include long-term follow-up studies *in vivo*. Compared to standard small molecular Gd-chelates, *GadofluorineM-Cy* has the distinct advantage of providing efficient cell labeling by simple incubation. Future studies will have to compare acute and long term MR imaging properties of stem cells labeled with *GadofluorineM-Cy* and alternative Gd-chelates.

In summary, this study presents a new approach for tracking hMSCs in cartilage defects based on *GadofluorineM-Cy* cell labeling as an alternative approach to SPIO-labeling. *GadofluorineM-Cy*-labeling provided significant MR signal effects of labeled stem cells, which remained stable through chondrogenic differentiation and thereby might be feasible for long term MASI monitoring. *GadofluorineM-Cy*, although not yet clinically approved, proved advantageous compared to SPIO due to less effect on chondrogenic differentiation and distinct contrast enhancement compared to postoperative artifacts from iron, hemorrhage or air.
